# A*Drosophila*genetic model of nephrolithiasis: transcriptional changes in response to diet induced stone formation

**DOI:** 10.1186/s12894-017-0292-5

**Published:** 2017-11-28

**Authors:** Vera Y. Chung, Benjamin W. Turney

**Affiliations:** Oxford Stone Group, Department of Urology, Nuffield Department of Surgical Sciences, University of Oxford, Oxford, UK

## Abstract

**Background:**

Urolithiasis is a significant healthcare issue but the pathophysiology of stone disease remains poorly understood. *Drosophila* Malpighian tubules were known to share similar physiological function to human renal tubules. We have used *Drosophila* as a genetic model to study the transcriptional response to stone formation secondary to dietary manipulation.

**Methods:**

Wild-type male flies were raised on standard medium supplemented with lithogenic agents: control, sodium oxalate (NaOx) and ethylene glycol (EG). At 2 weeks, Malpighian tubules were dissected under polarized microscope to visualize crystals. The parallel group was dissected for RNA extraction and subsequent next-generation RNA sequencing.

**Results:**

Crystal formation was visualized in 20%(±2.2) of flies on control diet, 73%(±3.6) on NaOx diet and 84%(±2.2) on EG diet. Differentially expressed genes were identified in flies fed with NaOx and EG diet comparing with the control group. Fifty-eight genes were differentially expressed (FDR <0.05, *p* < 0.05) in NaOx diet and 20 genes in EG diet. The molecular function of differentially expressed genes were assessed. Among these, Nervana 3, Eaat1 (Excitatory amino acid transporter 1), CG7912, CG5404, CG3036 worked as ion transmembrane transporters, which were possibly involved in stone pathogenesis.

**Conclusions:**

We have shown that by dietary modification, stone formation can be manipulated and visualized in *Drosophila* Malpighian tubules. This genetic model could be potentially used to identify the candidate genes that influence stone risk hence providing more insight to the pathogenesis of human stone disease.

## Background

Urolithiasis is a major healthcare problem accounting for over 83,000 hospital attendances in the UK each year [1]. There is a high (around 10%) lifetime prevalence of stone disease as well as recurrence rate worldwide [2]. In the last two decades, significant advance was made in the minimal invasive surgical treatment of stone disease. Effective medical therapies for prevention and treatment of stone disease are still lacking because little is known about the molecular pathophysiology of kidney stone disease.

Kidney stone is thought to be related to interaction of genetic factors and environment. In human renal stone disease, there are clear environmental influences including diet rich in animal protein, high salt intake, dehydration and obesity. Genetic predisposition to stone disease is also well recognized. Approximately 25% of stone formers have a positive family history [3] but only a small minority have a monogenic cause such as cystinuria, Dent’s disease or primary hyperoxaluria.

Overall, calcium oxalate (CaOx) stones constitute the most common (~80%) stone type and are currently regarded as idiopathic in etiology. A systematic genetic study of CaOx stones excluded monogenetic inheritance but twin studies have estimated the heritability of kidney stones to be 56% [4].

In order to study the pathogenesis of human CaOx stone disease, various animal models have been established. Amongst these, *Drosophila melanogaster* has been proposed as a powerful translational model of human nephrolithiasis [5]. The advantages include similarity between *Drosophila* Malpighian tubule and human renal tubule, cost-effectiveness, convenience to observe crystals and relative ease to perform genetic manipulation. The *Drosophila* Malpighian tubules share similar features with human renal tubules in terms of physiological function, anatomical structure and genetic activity [5]. Flies are an unprotected species and can be acquired and maintained at low cost. Their short life cycle allows rapid observation of the effects of environmental alternation. *Drosophila* has been shown to readily form CaOx stones with dietary supplement of lithogenic agents within days [6]. Furthermore, the *Drosophila* genome is highly conserved and fully characterized. Extensive genomic tools are readily available to facilitate genetic manipulation in this versatile model organism. As such, we have used *Drosophila* as a genetic model to try to better understand the molecular pathophysiology of stone disease.

## Methods

### *Drosophila*stocks

Wild-type *Drosophila melanogaster* (Canton S strain) were raised in standard fly medium (composition: 72.3% water, 18.6% maize, 3.8% yeast, 2.2% soya, 3.1% agar) in a 25 °C, 50–60% humidity incubator. Only male flies were selected for the experiment.

### Lithogenic diets

Three experimental diets were prepared: control group with standard fly medium, 0.05% Sodium oxalate (NaOx) added to standard fly medium and 0.5% ethylene glycol (EG) added to standard fly medium. Biological triplicates were prepared in each group.

### Fly lifespan study

New emergents were isolated on day 3 after eclosion under light anesthesia. They were randomly divided into 3 treatment groups. The starting population for each condition was 100. The maintenance density was 25 flies per vial. Every 3 days the flies were transferred to fresh medium and numbers of dead flies were counted. Lifespan was recorded and subjected to survival analysis.

### Malpighian tubules preparation

After 2 weeks of feeding on three different diets, the flies were killed on CO_2_ pad. The Malpighian tubules and hindgut were extracted in physiological medium (Schneider’s drosophila medium.

Fifteen flies were dissected for each biological triplicate. Freshly dissected tubules and hindgut were prepared for polarized light microscopy examination. Parallel groups were dissected and processed for RNA extraction.

### Birefringence microscopy

Malpighian tubules and hindgut were prepared fresh and observed under normal and polarized white light with an Olympus BX60 microscope. The percentage of crystal formation were documented and tubules were photographed. The tubules with crystal formation were photographed and scaled.

### RNA extraction and quality control

Total RNA was extracted from Malpighian tubules and hindgut of 15 flies in each biological replicate.

Qiagen MiRNeasy Mini Kit was used for RNA extraction.

RNA integrity was verified with Agilent 2100 Bioanalyzer (RNA 6000 Pico kit). Next generation RNA sequencing was performed and sequencing data was analyzed in collaboration with the Computational Biology Research Group using STAR (Spliced Transcripts Alignment to a Reference) and edgeR software.

### Statistics

Statistical analyses were carried out with Prism biostatistical software (Graphpad, San Diego, CA).

## Results

### Effect on lifespan

**Fig. 1:**
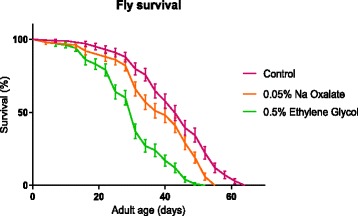
Drosophila survivorship in response to administration of lithogenic agents (0.05% NaOx, 0.5% EG) compared with control group. The lifespan was significantly reduced by food enriched with 0.05% NaOx (*p* < 0.0001) and 0.5% EG (p < 0.0001) compared with control group. EG had a more pronounced impact on the lifespan compared with NaOx (p < 0.0001). Log rank test was used to for comparison of survival curves

The lifespan was significantly reduced by food enriched with 0.05% NaOx and 0.5% EG (*p* < 0.0001) (Fig. 1). The administration of EG had a more pronounced impact on the lifespan compared with NaOx (*p* < 0.0001). The median and maximum lifespan in the control group was 43 and 64 days, 40 and 55 days in the NaOx diet, 31 and 52 days in the EG diet respectively.

### Inducing crystallization in Malpighian tubules

**Fig. 2:**
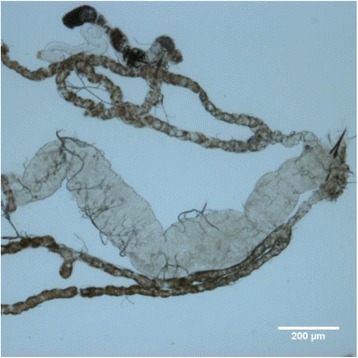
Photograph of Malpighian tubules under 10× s magnification. Two pairs of Malpighian tubules coalesce into a common ureter which empty into the junction of midgut and hindgut

The normal anatomy of Malpighian tubules is shown in Fig. 2. There are two pairs of Malpighian tubules in *Drosophila*: anterior and posterior pair. The two tubules joins into the ureter which then empties into the midgut and hindgut junction. One Malpighian tubule measures about 2 mm long, with the inner diameter measuring about 50 um. Urine was generated via transport of ions, solutes followed by water across the tubular lumen, and sequentially excreted to the hindgut [5].

The concentration of lithogenic agents (0.05% for NaOx and 0.5% for EG) was selected following optimization studies to adequately induce crystallization without leading to excessive premature death [6].

**Fig. 3:**
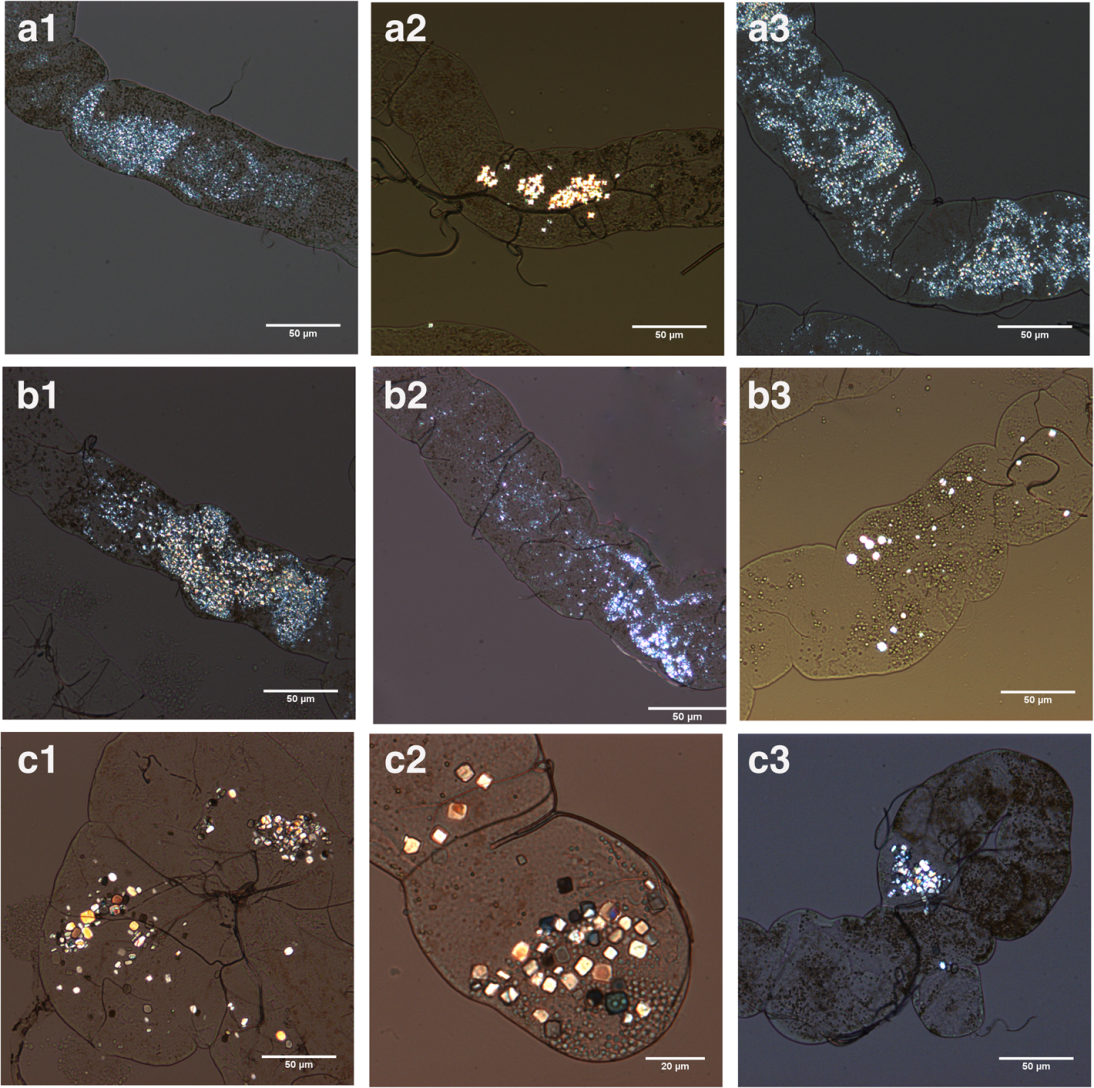
Degree of crystal formation in Malpighian tubules by visual scale, 20× s magnification. 0: no stone formation, +: mild, 2+: moderate, 3+ strong crystal formation

Crystal formation was detected as early as 1 week after initiation ingestion of lithogenic diets. Degree of crystallization was given a visual scale of 0, 1+, 2+, and 3+ in each condition (Fig. 3) [6]. All scoring was performed by a single observer.

The birefringent crystals were most commonly visualized in the distal tubules. There were no crystals observed in the hindgut.. It was noticed that the crystal morphology in the NaOx group was small and extensive, in contrast to the more sizable and polyangular shape in the EG diet.

**Fig. 4:**
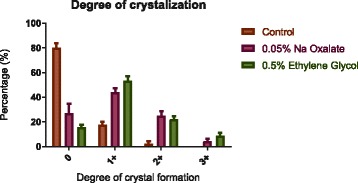
Incidence of crystal formation according to degree of crystallization. The degree of crystallization was given a visual scale of 0, 1+, 2+ and 3+ by a single observer. The incidence of crystal formation of the control group was 80%(0), 17.8% (1+) and 2.2% (2+) respectively. The incidence of crystal formation in flies fed on NaOx diet was 27% (0), 43.8% (1+), 25% (2+) and 4.2% (3+). In the EG cohort, the incidence was 15.6% (0), 53.3% (1+), 22.2% (2+), 8.9% (3+) respectively

There was significantly (*p* < 0.003 by chi-square test) higher rate of stone formation in the NaOx and EG diets (Table 1). Flies on control diet had a mean incidence of 20% stone formation whereas the incidence was 73% and 84% in NaOx and EG diets respectively. The incidence of crystal formation subcategorized to mild (1+), moderate (2+) and strong degrees (3+) was illustrated in Fig. 4.

**Table 1:** Incidence of stone formation in Drosophila Malpighian tubules in 3 experimental conditions

Drosophila diet	Overall incidence of crystal formation (%)
control	20 ± 2.2
0.05% sodium oxalate	73 ± 3.6
0.5% ethylene glycol	84 ± 2.2

Values were expressed as means ± standard error of mean

### RNA sequencing and quality control

**Fig. 5:**
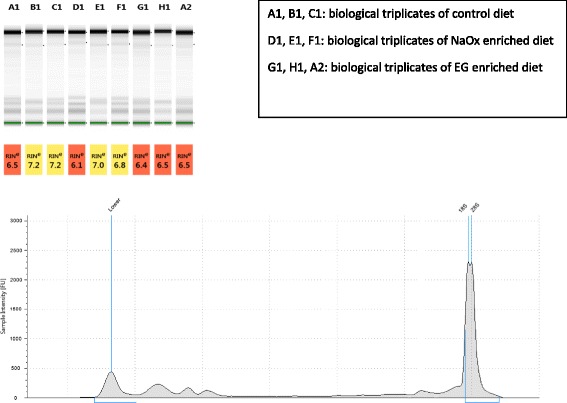
RNA quality verification by Agilent 2100 Bio-analyzer. Gel image and electrophoretic profile of *Drosophila* Malpighian tubules RNA samples showed high quality RNA extraction. The electrophoretic ribosomal RNA profile of insects has single 28S peak which is commonly seen in insect RNA

The parallel group of experimental flies was dissected after 2 weeks on lithogenic diets. Malpighian tubules were processed for total RNA extraction. Quality of extracted RNA was verified by Nanodrop and Agilent 2100 Bioanalyzer. The electrophoretic ribosomal RNA (rRNA) profile of insects was different from the standard rRNA integrity benchmark. It was common to observe single rRNA peak in insect RNA which does not represent degradation [7]. The electrophoretic profile in Drosophila RNA could be explained by the fact that the 28 s rRNA was processed into two fragments that co-migrate with the 18 s rRNA. The RNA integrity in our samples was shown to be satisfactory with tight banding and no evidence of degradation (Fig. 5).

After verification of RNA integrity, next generation RNA sequencing was performed to sequence the whole transcriptome of 9 samples. The mRNA was extracted from the total RNA and subsequently converted to cDNA. Second strand cDNA synthesis incorporated dUTP. The cDNA was end-repaired, A-tailed and adapter-ligated. Samples underwent uridine digestion and then amplification. The prepared libraries were size selected, multiplexed before paired end sequencing over one lane of a flow cell.

### Sequenced genes and possible candidate genes

**Fig. 6:**
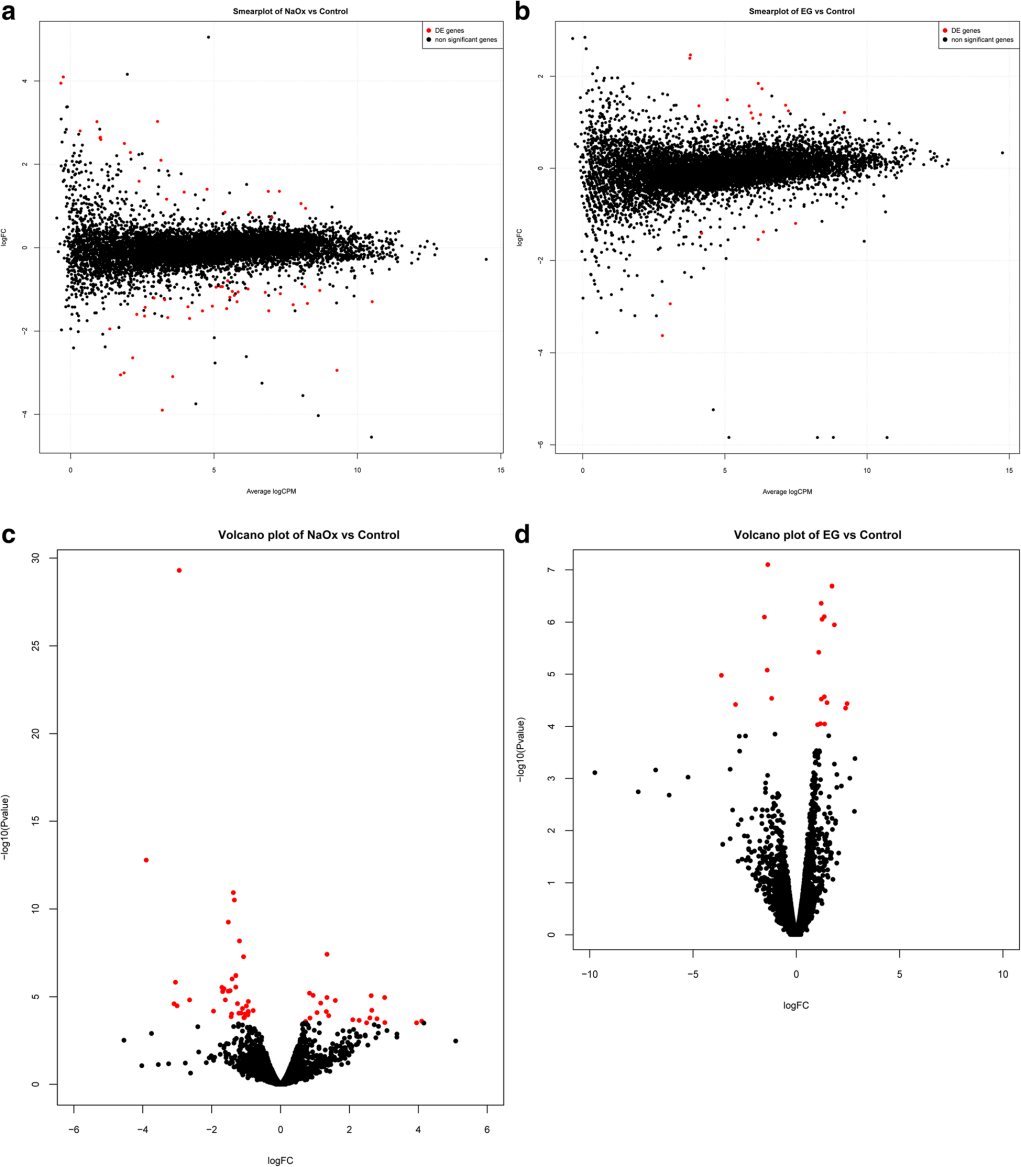
Smear plots and volcano plots of differentially expressed genes in NaOx and EG groups. Differentially expressed genes (FDR <0.05, *p* < 0.05) compared with control were labelled as red points whereas insignificant genes were labelled as black. The mean expression level, Log Fold Change and *p*-Value of differentially expressed genes were displayed as smear plots and volcano plots. **a** Smear plot of NaOx group compared with control. **b** Smear plot of EG group compared with control. **c** Volcano plot of NaOx group compared with control. **d** Volcano plot of EG group compared with control

**Fig. 7:**
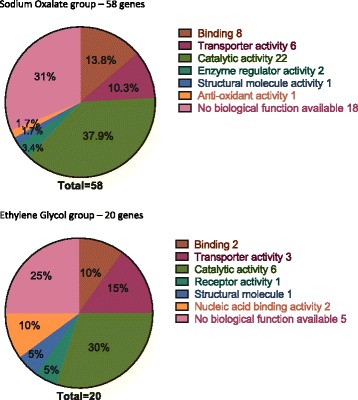
Molecular functions of differentially expressed genes

Library preparations were analyzed by the Computational Biology Research Group, University of Oxford, UK. Differentially expressed genes with FDR (false detection rate) < 0.05 and *p*-value <0.05 in all three biological triplicates were listed (Table 2). There were a total 58 genes identified in NaOx diet and 20 genes in EG diet. The mean expression level, Log Fold Change and *p*-Value of differentially expressed genes compared with control were displayed as smear plots and volcano plots (Fig. 6). Their molecular functions were matched in Flybase database and categorized (Fig. 7). Although there was no overlap between the differentially expressed genes in both treatment groups, their biological functions were very similar.

**Table 2:** Differentially expressed genes in NaOx and EG group compared with control

	Gene ID	Log Fold Change	*P* value	FDR
Sodium oxalate group (58 genes)	ymp CG4907 CG13806 CG15086 CG43755 w-cup CG3124 CG9975 CG9411 S-Lap7 CG4836 Muc68Ca Drs CG10383 Drsl2 CG12896 CG14695 CG6967 Cry CG13075 CG8678 CG5953 l(1)G0148 Slif Eaat1 Cyp28d1 CG4783 CG9238 Jon74E CG2781 CG1946 CG8083 mag zetaTry Npc2d CG42492 CG7912 Bace nrv3 CG10592 CG5150 CG32483 TotM CG15822 TotA Diedel3 CG12057 CG18607 CG6012 CG7781 TotC CG9134 CG6639 CG12374 CG34462 CG4830 CG34109 Obp56d	4.10 3.95 3.03 3.02 2.80 2.65 2.63 2.59 2.50 2.28 2.10 1.59 1.40 1.35 1.35 1.33 1.17 1.06 0.95 0.85 0.84 0.73 −0.79 −0.94 −0.94 −0.94 −0.95 −0.99 −1.03 −1.05 −1.06 −1.07 −1.11 −1.15 −1.19 −1.21 −1.25 −1.30 −1.30 −1.34 −1.37 −1.41 −1.42 −1.43 −1.46 −1.52 −1.52 −1.60 −1.64 −1.68 −1.70 −1.95 −2.64 −2.94 −3.00 −3.05 −3.09 −3.90	2.46E-04 3.08E-04 2.97E-04 1.10E-05 1.79E-04 5.88E-05 8.60E-06 1.60E-04 3.06E-04 2.21E-04 2.04E-04 1.62E-05 1.20E-04 3.85E-08 1.09E-05 6.96E-05 2.29E-05 7.99E-05 8.28E-06 1.63E-04 6.20E-06 2.58E-04 6.09E-05 1.85E-05 7.78E-05 6.84E-05 1.07E-04 3.32E-05 9.67E-05 1.54E-04 1.57E-04 5.32E-08 4.66E-05 8.53E-05 6.69E-09 8.67E-05 2.44E-05 6.24E-07 2.79E-06 3.06E-11 1.16E-11 9.60E-07 9.48E-05 1.36E-04 4.46E-06 5.61E-10 4.76E-06 1.50E-05 3.45E-06 5.05E-06 2.89E-06 6.57E-05 1.50E-05 5.04E-30 3.27E-05 1.47E-06 2.51E-05 1.61E-13	4.27E-02 4.99E-02 4.99E-02 4.69E-03 3.30E-02 1.67E-02 4.04E-03 3.07E-02 4.99E-02 3.92E-02 3.68E-02 6.09E-03 2.50E-02 5.17E-05 4.69E-03 1.77E-02 7.95E-03 1.92E-02 4.04E-03 3.07E-02 3.24E-03 4.40E-02 1.68E-02 6.67E-03 1.92E-02 1.77E-02 2.29E-02 1.01E-02 2.11E-02 3.07E-02 3.07E-02 6.24E-05 1.37E-02 1.99E-02 1.05E-05 1.99E-02 8.12E-03 6.51E-04 2.09E-03 7.20E-08 3.64E-08 9.01E-04 2.11E-02 2.79E-02 2.79E-03 1.05E-06 2.79E-03 5.89E-03 2.31E-03 2.79E-03 2.09E-03 1.76E-02 5.89E-03 4.73E-26 1.01E-02 1.26E-03 8.12E-03 7.56E-10
Ethylene glycol group (20 genes)	Hr38 Dh44 CG12057 CG17192 CR43314 Ets21C CG8299 CG18744 CG3036 CG1718 CG5404 CG31324 CG5151 W Jon66Ci Jon66Cii IM2 CG5770 Cpr49Ae LysP	2.46 2.38 1.84 1.73 1.49 1.37 1.36 1.35 1.25 1.21 1.20 1.16 1.09 1.03 −1.19 −1.38 −1.41 −1.54 −2.94 −3.63	3.68E-05 4.49E-05 1.12E-06 2.04E-07 3.53E-05 8.96E-05 2.72E-05 7.85E-07 8.81E-07 3.01E-05 4.35E-07 8.84E-05 3.80E-06 9.25E-05 2.92E-05 7.94E-08 8.36E-06 7.97E-07 3.83E-05 1.05E-05	2.22E-02 2.46E-02 1.49E-03 9.48E-04 2.22E-02 4.30E-02 2.15E-02 1.36E-03 1.36E-03 2.15E-02 1.35E-03 4.30E-02 4.42E-03 4.30E-02 2.15E-02 7.38E-04 8.64E-03 1.36E-03 2.22E-02 9.78E-03

Positive log FC (Fold changes) denoted up regulation of genes whereas negative Log FC denoted down regulation

*FDR* False detection Rate

We identified those genes which function as transmembrane ion transporter that may be actively involved in in crystal formation. These potential candidate genes and their specific molecular functions were displayed in Table 3.

**Table 3:** Candidate genes which function as transmembrane ion transporter, Ca ion binder or diuretic hormone that may play a role in crystal formation

	Gene name	GO Molecular function	Log FC
Sodium oxalate group	nervana 3	plasma membrane Na:K- exchanging ATPase activity	−1.29
	Excitatory amino acid transporter 1	Na: dicarboxylate symporter activity; Na:glutamate symporter activity	−0.93
	CG7912	High affinity Sulfate transmembrane transporter	−1.25
	Crystallin	Ca ion binding	0.945
Ethylene Glycol group	CG3036	Na: phosphate symporter activity	1.24
	CG5404	high affinity sulfate transmembrane transporter	1.2
	Dh44	Diuretic hormone activity	2.38

## Discussion

The formation of kidney stones is likely the result of complicated interactions of multiple genetic factors in addition to environmental influence. In this model we have successfully induced CaOx crystals in *Drosophila* Malpighian tubules by diets enriched with lithogenic agents. Our study is the first to try to elucidate transcriptive changes in response to stone formation in the *Drosophila* model.

A previous pilot study on the experimental induction of lithogenic agents in *Drosophila* has shown that such diets had a negative impact on *Drosophila* survival in a dose dependent manner [6]. The crystal composition was analyzed by energy-disperse X-ray spectroscopy and scanning electron microscopy, which revealed predominantly CaOx monohydrate or CaOx dihydrate [6, 8].

Our survival study demonstrated in similar fashion that *Drosophila*’s lifespan was significantly reduced by food enriched with 0.05% NaOx and 0.5% EG. The overall incidence crystal formation in control flies in our experiment was higher (20% vs 6.6%) but slightly lower in the lithogenic diets (84% vs 94%) compared with the pilot study.

Unlike the mammalian species, the Malpighian tubule in insects is the only organ for excreting solutes and water to the tubular lumen, as there is no glomerulus [9]. The distal segment of Malpighian tubules secretes electrolytes, organic solutes and water, and the proximal segments function as solute and water reabsorption [10]. The body calcium levels was regulated by Malpighian tubules by excretion through secretion into the lumen [11]. Administration of NaOx provides additional dietary supplement of oxalate, little evidence is available on how EG was metabolized in *Drosophila* however. Inferred from the metabolic pathway in the mammals, EG is degraded into four components: glycoaldehyde, glycolic acid, glyoxylic acid and oxalic acid. Excessive oxalic acid precipitates as CaOx stone in the tubules as a result [12]. The survival study demonstrated that the crystal accumulation in Malpighian tubules has a negative impact on the *Drosophila* lifespan, probably as a result of obstruction to the excretory organs and subsequent inability to maintain body hemostasis. EG induced a more profound negative impact on the lifespan, which could be related to associated metabolic acidosis [12] due to accumulated glycolic acid in addition to crystal formation.

Among the 58 differentially expressed genes in NaOx treatment group and 20 in EG group, we narrowed down the potential candidate genes by a number of criteria: molecular function, biological process and Log Fold changes compared with expression levels in the control group. There were no functionally significant overlapping genes between the two treatment groups, probably due to the fact that different biological pathways were involved in NaOx and EG diets. Genes that functions as transmembrane ion transporters had been singled out, namely Nervana 3, CG7912 and Eaat 1 in NaOx group; CG3036 and CG5404 in EG group. Crystallin coding for calcium ion binding protein and Dh44 which functions a diuretic hormone involved in body fluid secretion might also be of interest.

Nervana 3 codes for β subunit of Na+/K+ transporting ATPase, which catalyzes the hydrolysis of ATP coupled with the exchange of Na + and K+ ions across the plasma membrane. The β subunit regulates the transportation of sodium pumps to the plasma membrane [13]. Na + and K+ transport has been implicated in Ca2+ ion across renal tubules and homeostasis [14]. Though there was no previous work to elucidate the role of Na+/K+ pump in the Malpighian tubule, it was shown in rat and rabbit model that the basal-lateral plasma membrane contain Na+/Ca2+ exchange system which mediates the counter-transport of Ca2+ and Na + across the basal cell border [15, 16]. A protein which functioned as Na+/Ca2+/K+ exchanger has been described in Drosophila to promote Ca2+ extrusion from cells [17].

CG7912 is predicted to have the molecular function of high-affinity sulfate transmembrane transporter. Its function in *Drosophila* was inferred from homology to mammalian transporters. Presumably, it catalyzes the high affinity transfer of sulfate across the plasma membrane up its concentration gradient ([18] Flybase). In the murine model, targeted disruption of renal sulfate transporter genes NaS1 and Sat1 leads to hyposulfatemia and hypersulfaturia. Loss of Sat1 leads to hyperoxaluria, hyperoxalemia and calcium oxalate urolithiasis [19, 20]. These data suggest that sulfate transporters play an essential role for sulfate and oxalate homeostasis.

Similarly, the function of excitatory amino acid transporter (Eaat 1) as a sodium dicarboxylate symporter is inferred from homology to mammalian transporters. Its coding protein is an integral component of membrane which mediates concomitant uptake of sodium and dicarboxylate [21]. The rat ortholog of sodium dicarboxylate transporter (NaDC) was reported to mediate citrate uptake from renal proximal tubule. Citrate is an important inhibitor of calcium-stone formation and most of the citrate reabsorption is thought to occur via a sodium dicarboxylate transporter (NaDC1) located in the apical membrane. NaDC1 has been localized in opossum kidney proximal tubule cells and was responsible for calcium regulated citrate reabsorption in proximal tubules [22]. In the rat model, increased NaDC1 expression on the renal proximal tubule epithelial cells was associated with a decline in urinary citrate excretion, suggesting this transporter could play an important role in nephrolithiasis development [23]. In our study, Eaat1 demonstrated decreased expression in sodium oxalate diets compared with control, suggesting a protective effect to tubular stone formation.

The other candidate gene, Crystallin encodes a calcium ion binding protein [24] and is a major structural constituent of eye lens [25]. The implication of this gene in the biological process of stone formation is yet to be elucidated.

Similarly, in the EG treatment group, the protein products of gene CG3036 and CG5404 work as transmembrane transporter i.e. sodium-phosphate symporter and high affinity sulfate transmembrane transporter, despite that the function of these gene products has not been directly measured Sodium phosphate co-transporter (Npt2a) null mice are shown to have hypercalciuria and hyperphosphaturia, and develop tubular and interstitial Ca phosphate deposits [26]. Crystals of CaOx may result for nucleation surrounding the Ca phosphate crystal [27] In human, hypophosphatemic hypercalciuric nephrolithiasis associated with rickets is the related to mutations in the type 2c sodium-phosphate co-transporter [28]. The up regulation of CG3036 in Drosophila might be a counteractive response to lower down tubular Ca level to reduce CaOx stone formation.

Dh44 is a gene that encodes a corticotrophin releasing factor (CRF) like peptide with 44 amino acids. Functional studies showed that the protein product stimulated fluid production, and this effect was mediated by cyclic AMP in principal cells only [29]. The significant up regulation of this diuretic hormone in Malpighian tubules could be a direct protective effect of body fluid secretion in response to crystal formation.

These differentially expressed genes could play a role in the biological pathway of calcium oxalate stone formation or could be a regulated response to the obstructive uropathy, metabolic acidosis and failure to maintain electrolyte or acid-base balance in *Drosophila* Malpighian tubules. We made the hypothesis that genes encoding transmembrane ion transporter or diuretic hormone might be more critical in these processes but other genes might also be relevant. Molecular function of many differentially expressed genes remained unknown, so that further investigations will be required to elucidate the exact roles of these genes and how they function as a whole in the process of CaOx crystal formation.

Despite the high incidence of crystal formation in flies fed with lithogenic agents, we do not know if there was any difference in the transcriptive changes in stone formers and non-stone former. Experiments designed to study the difference of stone-formers and non-stone formers might provide further insight in the pathogenesis.

In this experiment of environmentally induced stone formation, we have successfully used the drosophila as a model to demonstrate transcriptional changes in a number of genes that may play a role in the pathogenesis of stone formation. To evaluate the contribution of these genes to stone formation, RNAi could be used to preform tissue-specific knockdown or overexpression studies to modify stone formation, using the extensive collection of genetic reagents available in *Drosophila*. Downstream proteomic analysis could further provide information of the function of possible candidate genes.

## Conclusions

Our results suggest that drosophila proved to be a powerful experimental organism for studying nephrolithiasis. The transcriptome secondary to crystal formation could provide useful insight into the pathophysiology of stone formation and potential therapeutic target for the treatment of nephrolithiasis.

## Abbreviations

CaOx: Calcium oxalate
EG: Ethylene glycol
FC: Fold changes
FDR: False discovery rate
mRNA: Messenger RNA
NaOx: Sodium oxalate
rRNA: Ribosomal RNA
